# Supplementary figures and images for: Integrated Transcriptomic and Proteomic Characterization of a Chromosome Segment Substitution Line Reveals the Regulatory Mechanism Controlling the Seed Weight in Soybean
Source: Plants (Basel). 2024 Mar 21;13(6):908. doi: 10.3390/plants13060908 (PMC10975824; doi:10.3390/plants13060908)

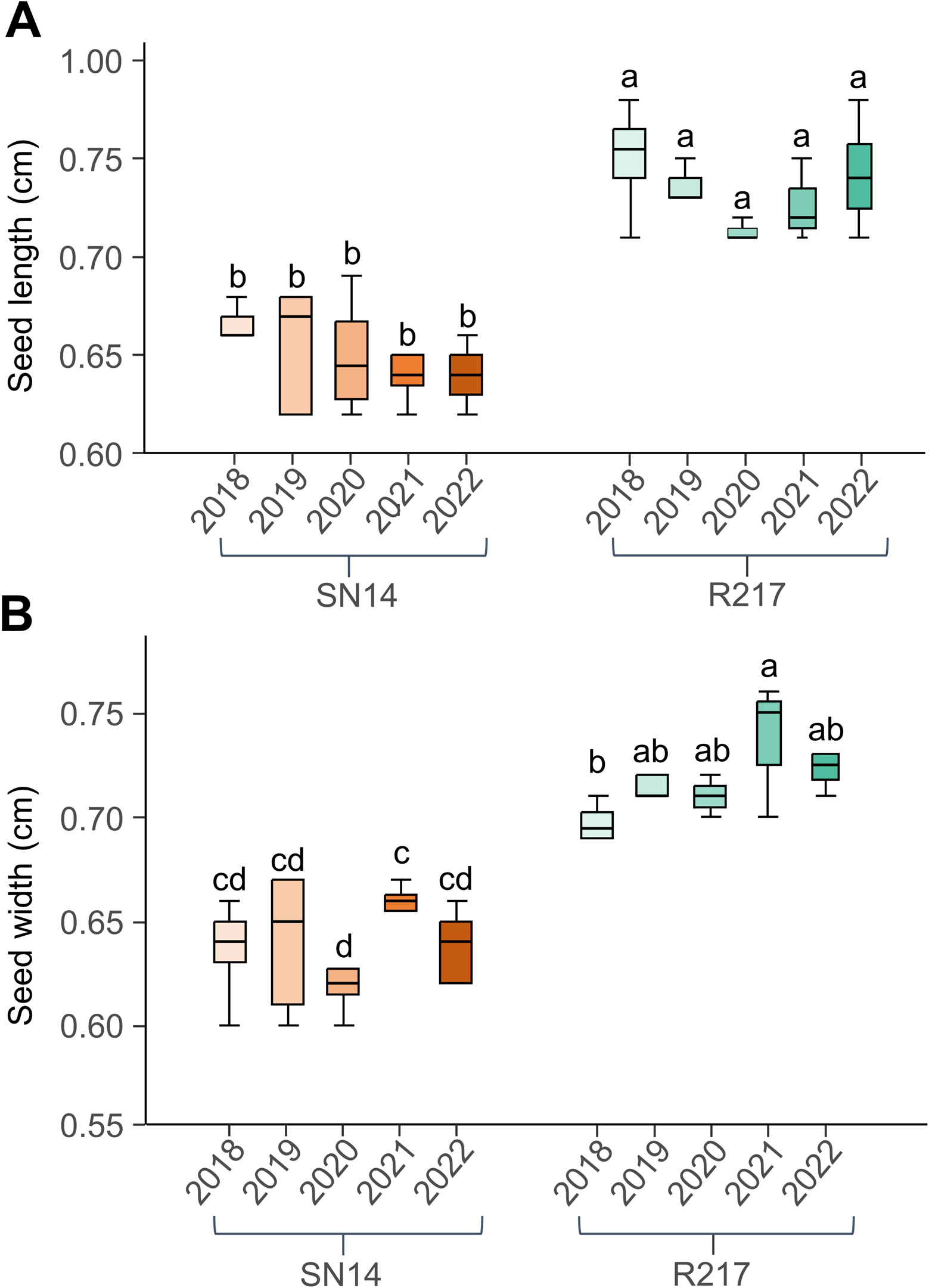

Supplement: Supplementary file 1 [file plants-13-00908-s001.zip › Figure.S1.jpg]

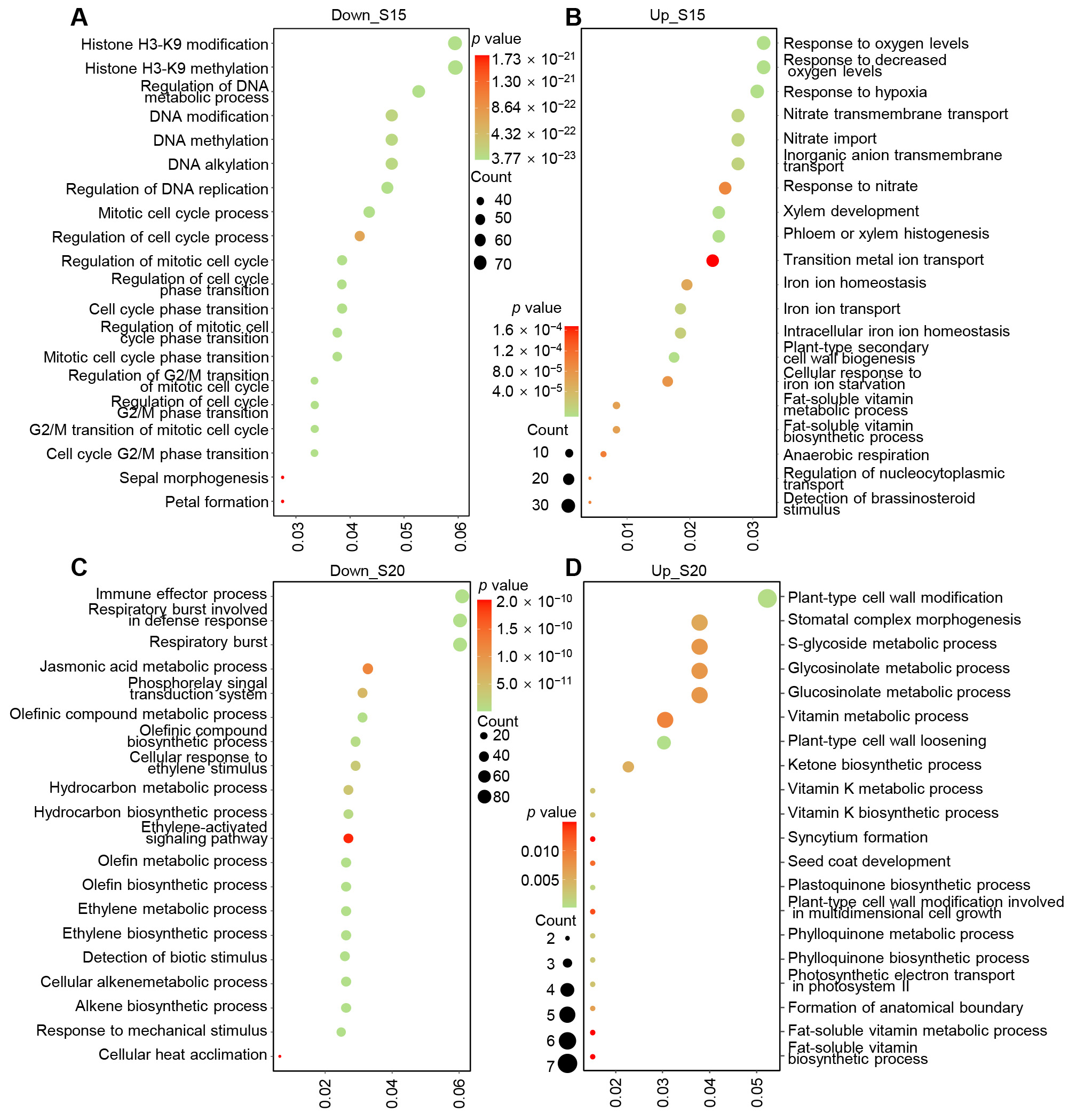

Supplement: Supplementary file 1 [file plants-13-00908-s001.zip › plants-SupplementaryFigure2-final_version20240206.jpg]
